# Versorgungssituation von Parkinson-Patienten in Sachsen: Eine sekundärdatenbasierte Analyse der Inanspruchnahme im Beobachtungszeitraum 2011 bis 2019

**DOI:** 10.1007/s00115-022-01273-7

**Published:** 2022-03-14

**Authors:** Patrick Timpel, Falko Tesch, Gabriele Müller, Caroline Lang, Jochen Schmitt, Peter Themann, Ute Hentschker-Ott, Björn Falkenburger, Martin Wolz

**Affiliations:** 1 Zentrum für Evidenzbasierte Gesundheitsversorgung, Universitätsklinikum und Medizinische Fakultät Carl Gustav Carus, Technische Universität Dresden, Fetscherstraße 74, 01307 Dresden, Deutschland; 2 Fachbereich Neurologie/Parkinson, Klinik am Tharandter Wald, Hetzdorf, Deutschland; 3 Deutscher Bundesverband für akademische Sprachtherapie und Logopädie, Moers, Deutschland; 4 Klinik und Poliklinik für Neurologie, Universitätsklinikum Carl Gustav Carus, Technische Universität Dresden, Dresden, Deutschland; 5 Klinik für Neurologie und Geriatrie, Elblandklinikum Meißen, Meißen, Deutschland

**Keywords:** Sekundärdatenanalyse, Inanspruchnahme, Neurologie, Medikamentöse Parkinsontherapie, Heilmittelerbringung, Secondary data-based analysis, Utilisation, Neurology, Pharmacological treatment, Provision of medical remedies

## Abstract

**Hintergrund:**

Als Bundesland mit dem höchsten Altersdurchschnitt in Deutschland und besonderen Strukturmerkmalen ländlich geprägter Gebiete sind die Folgen des demographischen Wandels bereits heute in Sachsen spürbar. Um die medizinische Versorgung von Parkinson-Patienten zu verbessern, bedarf es einer Status-quo-Analyse der aktuellen Versorgungspraxis.

**Ziel der Arbeit (Fragestellung):**

Inwieweit unterscheidet sich die Inanspruchnahme der medizinischen Leistungserbringung von Parkinson-Patienten im Vergleich von städtisch und ländlich geprägten Gebieten sowie im Vergleich von Parkinson-Patienten mit und ohne Neurologenkontakt im Beobachtungszeitraum von 2011 bis 2019?

**Material und Methoden:**

Die Kohortenstudie basiert auf umfangreichen Routinedaten der Krankenkasse AOK PLUS der Jahre 2010 bis 2019 für Sachsen. Untersucht wurde eine Kohorte von insgesamt 15.744 Parkinson-Patienten (*n* = 67.448 Patientenjahre) und eine gematchte Vergleichskohorte (*n* = 674.480 Patientenjahre; Kriterien: Geburtsjahr, Geschlecht, Versicherungsjahr, Wohnsitz Stadt/Land) ohne ICD-10-Kodierung einer Bewegungsstörung.

**Ergebnisse:**

Insgesamt war eine kontinuierliche Zunahme der Anzahl der Erkrankten in der dynamischen Kohorte von 2011 (*n* = 6829) bis 2019 (*n* = 8254) zu beobachten. Stadt-Land-Unterschiede zeigten sich insbesondere in der geringeren (Mit‑)Behandlung durch niedergelassene Neurologen in ländlich geprägten Gebieten. Parkinson-Patienten hatten ein 3,5- bzw. 4‑fach erhöhtes Risiko zu versterben im Vergleich zu Versicherten der Vergleichskohorte. Veränderungen der medikamentösen Parkinson-Therapie (Zunahme COMT- und MAO-Inhibitoren) sowie der Heilmittelerbringung (Zunahme Ergotherapie und Logopädie) über die Beobachtungszeit zeigten sich primär bei Parkinson-Patienten mit Neurologenkontakt.

**Diskussion:**

In der Studie konnten eine erhöhte Morbidität und Mortalität bei Parkinson-Patienten identifiziert werden, die sich als Ziel für innovative Versorgungskonzepte eignen. Die zunehmende Zahl an Patienten und die beschriebenen Unterschiede dokumentieren hierfür den Bedarf. Gleichzeitig zeigen die Veränderungen in der Verordnungspraxis, dass innovative Therapien von niedergelassenen Neurologen eingesetzt werden.

**Zusatzmaterial online:**

Die Onlineversion dieses Beitrags (10.1007/s00115-022-01273-7) enthält zusätzliche Tabellen und Infomaterialien. Beitrag und Zusatzmaterial stehen Ihnen auf www.springermedizin.de zur Verfügung. Bitte geben Sie dort den Beitragstitel in die Suche ein, das Zusatzmaterial finden Sie beim Beitrag unter „Ergänzende Inhalte“.

## Abstract (en)

**Background:**

The consequences of demographic change are already noticeable in Saxony, the federal state with the highest average age in Germany and predominantly rural areas. In order to improve medical care for patients with Parkinson’s disease (PwP), a status quo analysis of current care practice is required.

**Objective:**

To what extent does the utilization of medical services by PwP differ a) between urban and rural areas in Saxony and b) between PwP with and without neurologist contact in the observation period from 2011 to 2019?

**Material and methods:**

The cohort study was based on extensive routine data for Saxony from the health insurance company AOK PLUS from 2010 to 2019. A cohort of 15,744 PwP (*n* = 67,448 patient-years) was compared to a matched cohort (*n* = 674,480 patient-years; criteria: year of birth, gender, year of insurance, place of residence: urban/rural) without an ICD-10 coding of a movement disorder.

**Results:**

Overall, there was a steady increase in the number of PwP in the dynamic cohort from 2011 (*n* = 6829) to 2019 (*n* = 8254). Urban-rural differences included a smaller proportion of patients being seen by a neurologist in rural areas. The PwP had a 3.5 to 4‑fold higher risk of dying compared to those in the comparison cohort. Changes in drug therapy for Parkinson’s disease (i.e., increases in COMT and MAO inhibitors) and in remedy delivery (i.e., increases in occupational therapy and speech therapy) over the observation period were primarily seen in PwP who were seen by a neurologist.

**Discussion:**

The study identified increased morbidity and mortality in PwP who are suitable targets for innovative care concepts. The increasing number of patients and the described differences document the need for this. At the same time, changes in prescription practice show that innovative forms of treatment are being used by neurologists in outpatient care.

Patienten^1^ mit neurologischen Erkrankungen, wie dem Morbus (Mb.) Parkinson, benötigen individualisierte und komplexe Versorgungskonzepte unter Beteiligung von Spezialisten. Die steigende Prävalenz und Unterschiede in den regional verfügbaren Versorgungsstrukturen erfordern eine regionale Analyse der aktuellen Versorgungssituation. Der vorliegende Beitrag untersucht die Inanspruchnahme der medizinischen Leistungserbringung von Parkinson-Patienten in Sachsen im Vergleich von städtisch und ländlich geprägten Gebieten im Beobachtungszeitraum von 2011 bis 2019 im Rahmen einer Sekundärdatenanalyse.

## Hintergrund

Neurologische Erkrankungen sind weltweit eine Hauptursache für in Krankheit verbrachte Lebensjahre. Dabei hat sich die Prävalenz der Parkinson-Patienten zwischen 1990 und 2016 mehr als verdoppelt [11]. Für Deutschland wurde die Parkinson-Prävalenz 2016 auf 162.246 Patienten geschätzt [11]. Bedingt durch die steigende Lebenserwartung und das mit fortschreitendem Lebensalter ansteigende Erkrankungsrisiko wird erwartet, dass diese auch in Zukunft weiter ansteigen wird [10, 25].

Im Gegensatz zu anderen neurodegenerativen Erkrankungen sind die Symptome der Parkinson-Krankheit medikamentös und nichtmedikamentös sehr gut behandelbar [4, 8, 24, 28]. Der dafür erforderliche rechtzeitige, effiziente und altersgerechte Zugang zu Parkinson-spezifischen Therapiemöglichkeiten ist jedoch an regionale Versorgungsstrukturen gebunden [29].

Die wissenschaftliche Evaluation von Verordnungsdaten ist geeignet, um verschiedene versorgungsrelevante Aspekte, wie die Inanspruchnahme von Therapien oder aufgetretene Komplikationen großer Kohorten unter Routinebedingungen, zu analysieren und um Ansatzpunkte für innovative Versorgungskonzepte zu entwickeln [14, 27]. Die Daten aktuell verfügbarer Sekundärdatenanalysen zur Untersuchung der Inanspruchnahme medizinischer Leistungen von Parkinson-Patienten sind inzwischen veraltet (1991 bis 1997 [30]; 2004 bis 2009 [2]; 2004 bis 2010 [20]) oder beschränken sich auf eine Analyse der stationären Leistungserbringung [29]. Fehlende Analysen der ambulanten Leistungserbringung, oder auch der Hilfsmittelversorgung, erschweren bisher eine umfassende Abschätzung des sektorenübergreifenden Versorgungs- und Unterstützungsbedarfs. Der prognostizierte Anstieg der Parkinson-Patienten bis 2040 [9] und die Heterogenität der Versorgungsstrukturen, insbesondere im Vergleich städtisch und ländlich geprägter Gebiete, lassen jedoch Herausforderungen im Hinblick auf eine flächendeckende und qualitativ hochwertige Langzeitversorgung vermuten [26]. Neben einer regionalen Analyse der Versorgungssituation werden demnach ebenso Zeitreihen benötigt, um Veränderungen in der Inanspruchnahme beschreiben zu können [13, 20].

Ziel dieser Studie ist es daher, zu untersuchen, inwieweit sich die Inanspruchnahme der medizinischen Leistungserbringung von Parkinson-Patienten im Vergleich von städtisch und ländlich geprägten Gebieten sowie im Vergleich von Parkinson-Patienten *mit *und *ohne* neurologischen Facharztkontakt unterscheidet und wie sich die Versorgung im Beobachtungszeitraum von 2011 bis 2019 entwickelt. Die vorliegende Sekundärdatenanalyse ist Teil des Projektes *ParkinsonNetzwerk Ostsachsen *(PANOS)^2^ und analysiert den Status quo in Sachsen vor Durchführung einer prospektiven, kontrollierten Interventionsstudie zur Evaluation eines intersektoralen, pfadbasierten und plattformunterstützten Versorgungskonzeptes [17].

## Material und Methoden

### Studiendesign und Datenquellen

Datenbasis der Kohortenanalyse bildeten umfangreiche Routinedaten der Gesetzlichen Krankenversicherung (GKV) AOK PLUS der Jahre 2010 bis 2019 für Sachsen. In Deutschland sind ca. 90 % der Bevölkerung bei einer GKV versichert. Dabei sind im Freistaat Sachsen etwa die Hälfte der 4 Mio. Einwohner bei der AOK PLUS versichert [1]. Vor der Durchführung der Analysen wurde das Gesamtvorhaben durch die Sächsische Landesärztekammer positiv beschieden (EK-BR-117/20-1).

### Auswahlkriterien und Analyseeinheit

Durch vergleichbare Sekundärdatenanalysen [2, 13, 20, 23, 30] und den iterativen Austausch mit klinisch tätigen Konsortialpartnern wurden Aufgreifkriterien definiert und mittels Plausibilitätsanalysen überprüft. Versicherte wurden für das Folgejahr eingeschlossen, wenn sie alle drei Aufgreifkriterien innerhalb eines Jahres erfüllten (Tab. 1). Erkrankte mit Medikationspausen für einzelne Jahre wurden in Folge der internen Validierung (Plausibilisierung) ausgeschlossen.

**Table 1:** 

Kriterium	Beschreibung
1. Versichertenstatus	Gesetzlich krankenversichert bei der AOK PLUS zwischen 2010 und 2019 (keine minimale Versicherungszeit zu erfüllen)
2. Parkinson-Diagnose	≥ 2 ambulante Parkinson-Diagnosen (ICD-10 G20, M2Q)
	*ODER*
	≥ 1 stationäre Parkinson-Diagnose (ICD-10 G20)
3. Parkinson-spezifisches Medikament	≥ 1 Verschreibung eines Parkinson-spezifischen Medikaments (ATC-Codes siehe Zusatzmaterial 1)

*ICD-10* International Classification of Disease, 10th revision, *ATC* anatomisch-therapeutisch-chemische Klassifikation, *M2Q *mindestens 2 Quartale

### Vergleichsgruppen

Der Wohnsitz der Erkrankten wurde auf Basis der verfügbaren dreistelligen Postleitzahl (PLZ) stichtagsbezogen ermittelt, sodass eine Einteilung in Stadt (definiert als die drei Großstädte Dresden, Leipzig, Chemnitz; und deren Umland) und Land vorgenommen werden konnte. Zudem wurden Erkrankte *mit* im Vergleich zu jenen *ohne* Neurologenkontakt innerhalb einer Jahresscheibe gegenübergestellt.

Für die eingeschlossenen Parkinson-Patienten wurden jeweils 10 Versicherte mit Zurücklegen *ohne* ICD-10-Kodierung aus dem Indikationsgebiet *Extrapyramidale Krankheiten und Bewegungsstörungen (G20-G26) *zur Bildung einer Vergleichsgruppe ausgewählt. Das Matching erfolgte exakt auf den folgenden Merkmalen: Geburtsjahr, Geschlecht, Versicherungsjahr sowie Wohnsitz in städtisch oder ländlich geprägten Regionen Sachsens. Es wurden Merkmale aus der ambulanten und stationären Versorgung, der Versorgung mit Medikamenten, Heil- und Hilfsmitteln, dem Auftreten Parkinson-spezifischer Komplikationen sowie Merkmale der Morbidität und des Pflegebedarfs der Versicherten herangezogen.

### Statistische Analysen

Die Datenanalyse erfolgte deskriptiv. Dabei wurde die Zeit, welche die eingeschlossenen Parkinson-Patienten zur Kohorte beitrugen, als *Patientenjahre *ausgewiesen und für die untersuchte Gruppe aufsummiert. Dazu wurden Häufigkeitsverteilungen, Anteile oder – im Falle der zu berücksichtigenden Versicherungszeit/-wechsel und Tod – Raten berechnet. Um eine Überschätzung der Inanspruchnahme, insbesondere bei Erkrankten mit verminderter Versicherungszeit innerhalb eines Jahres, zu vermeiden, wurden bei Raten stets die obersten 1 % der Patienten mit vorhandenem Wert ausgeschlossen. Zudem wurden Kontingenztabellen – als tabellarische Darstellung der Häufigkeit zweier Merkmale – verwendet, um Versorgungsmerkmale zu vergleichen.

Für die analytische Betrachtung wurden Determinanten für mögliche Komplikationen der Parkinson-Krankheit (u. a. Hospitalisierungen, Pneumonie, Frakturen, Obstipationen, Halluzinationen) in einer Poisson-Regression mit „log“-Linkfunktion und robusten Standardfehlern über neun Jahre und einem „Offset“ für die Versicherungszeit verwendet.

Die Datenaufbereitung und Analyse erfolgte mittels IBM SPSS Statistics for Windows, Version 27.0 (IBM Corp, Armonk, NY, USA).

## Ergebnisse

### Deskription der Kohorte

Insgesamt wurde eine kontinuierliche Zunahme der Erkrankten mit Mb. Parkinson (in Patientenjahren) in der dynamischen Kohorte von 2011 (*n* = 6829) bis 2019 (*n* = 8254) beobachtet. Bezogen auf die Altersklasse der über 70-Jährigen stieg der Anteil der Parkinson-Patienten an der Gesamtzahl der Versicherten der AOK PLUS in Sachsen im gleichen Zeitraum um 28 %. Die Erkrankten wurden in den neun Jahren im Durchschnitt 4,3 Jahre lang beobachtet. Die identifizierten Parkinson-Patienten waren durchschnittlich 78,6 (SD 9,0) Jahre alt und lebten mehrheitlich in ländlich geprägten Gebieten (Tab. 2). Dabei hatten ca. 20 % keinen jährlichen Kontakt bei einem ambulanten Neurologen. Diese waren durchschnittlich älter und zu einem größeren Anteil pflegebedürftig als jene mit Kontakt (siehe *Zusatzmaterial 2, eTab. 1*).

**Table 2:** 

Soziodemographie	Stadt		Land		Insgesamt
	Ohne NK	Mit NK	Ohne NK	Mit NK	
Patientenjahre	3389	18.277	9891	35.891	67.448
Alter (Jahre)	81,2	78,7	81,0	77,6	78,6
Männeranteil (%)	41,3	47,4	41,3	48,7	46,9

*NK* Neurologenkontakt im jeweiligen Jahr

### Referenz zur Vergleichskohorte

#### Deskriptiver Vergleich

Insgesamt legt der Vergleich der dynamischen Parkinson-Kohorte mit einer 1:10-gematchten Vergleichskohorte eine erhöhte Morbiditätslast der Parkinson-Patienten nahe, z. B. in Form eines deutlich höheren Anteils an Erkrankten mit mindestens fünf dauerhaft eingenommen Medikamenten oder Pflegegrad/-stufe.

In rund 50 % der Patientenjahre wurde ein Hilfsmittel verschrieben. Für sechs der untersuchten acht Hilfsmittelbereiche zeigt sich ebenso eine gesteigerte Inanspruchnahme in der Parkinson-Kohorte.

Auch die Mortalität ist bei den Parkinson-Patienten höher als in der Vergleichskohorte. Innerhalb der Parkinson-Kohorte zeigt sich darüber hinaus eine deutlich erhöhte Mortalität bei den Erkrankten ohne Neurologenkontakt.

Während in der Parkinson-Kohorte hochgerechnet auf 100 Patientenjahre, knapp jede(r) Zweite (46,0 %) im Krankenhaus versorgt wurde, waren es bei der Vergleichskohorte lediglich knapp ein Drittel (Stadt: 29,6 %; Land: 31,0 %). Bei den a priori definierten Parkinson-spezifischen Komplikationen zeigt sich bei der Parkinson- im Verhältnis zur Vergleichskohorte eine Häufung von Harnwegsinfekten, Frakturen und Pneumonien. Krankenhausaufnahmen und -notaufnahmen wegen der Parkinson-Krankheit waren jeweils anteilig häufiger bei Erkrankten mit Neurologenkontakt (Tab. 3; *Zusatzmaterial 2, eTab. 3*).

**Table 3:** 

Merkmal	Parkinson-Kohorte (*n* = 67.448^a^)		Vergleichskohorte (*n* = 674.480^a^) (%)
	Ohne NK (*n* = 13.280) (%)	Mit NK (*n* = 54.168) (%)	
*Morbidität*			
Anzahl Dauermedikamente (MW (SD))	6,7 (3,7)	6,8 (3,5)	4,4 (3,5)
Anteil Polypharmazie (≥ 5 Medikamente)	71,9	73,1	43,2
MultiCare (0–44) (MW (SD))	9,9 (4,6)	10,2 (4,7)	8,7 (4,7)
Anteil mit Pflegestufe/-grad	60,7	53,1	21,2
*Hilfsmittel*			
Selbstversorgung u. Haushaltsführung	4,9	3,8	1,5
Pflegebetten	10,5	7,7	2,4
Kommunikation	3,7	4,2	4,5
Pflegerische Hilfsmittel	4,4	4,1	2,8
Toiletten- und Inkontinenzhilfen	14,1	13,0	5,6
Mobilität	31,1	36,7	22,4
Bewegungs- und Schmerztherapie	1,0	1,8	1,2
Körperhygiene	12,1	12,7	5,2
Insgesamt (unabhängig von der Kategorie)	53,0	54,3	33,8
*Komplikationen*			
Tod	17,9	10,9	6,2
Krankenhausaufnahmen	44,7	46,4	30,5
Krankenhausaufnahmen^b^	3,7	9,0	–
Krankenhausaufnahmen (Notfall)^b^	1,5	2,6	–
Harnwegsinfekt	9,1	8,9	5,6
Alle Frakturen	4,3	4,2	2,4
Oberschenkelhalsfraktur	1,0	0,8	0,3
Pneumonie ambulant	2,9	2,2	1,1
Pneumonie stationär	3,8	3,2	1,2
Obstipation/Ileus	1,5	1,2	0,6
Halluzinationen	0,4	0,7	0,02

Angaben beziehen sich auf Merkmalsausprägungen pro Kalenderjahr im Beobachtungszeitraum 2011 bis 2019; das Matching der Vergleichskohorte erfolgte entsprechend Alter, Geschlecht, Versicherungsjahr (2011 bis 2019) sowie Stadt/Land

*MultiCare* Liste an multimorbiditätsspezifischen Erkrankungen, *MW* Mittelwert, *n* Anzahl (absolute Zahlen), *NK* Neurologenkontakt im jeweiligen Jahr,* SD* Standardabweichung

^a^Angabe in Patientenjahren

^b^Merkmale beziehen sich auf Hospitalisierungen wegen Mb. Parkinson; Details siehe *Zusatzmaterial 2, eTab. 2 und 3*

#### Analytischer Vergleich

Die Ergebnisse der Regressionsanalysen (*Zusatzmaterial 2, eTab. 7*) zeigen, dass Männer im Vergleich zu Frauen der jeweiligen Altersgruppe ein erhöhtes Mortalitätsrisiko hatten (RR = 1,52 [95 %-CI 1,49–1,54]), jedoch seltener von Frakturen (RR = 0,58 [95 %-CI 0,56–0,60]) und Harnwegsinfektionen (RR = 0,61 [95 %-CI 0,59–0,62]) betroffen waren. Mit steigendem Alter nahmen die Mortalität und Komplikationen wie Frakturen (insbesondere Oberschenkelhals) und Pneumonien zu. Eine zunehmende Morbidität – gemessen an der Anzahl parallel vorliegender Erkrankungen – erhöhte das relative Risiko (RR) für Obstipation/Ileus und stationär behandelte Pneumonien. Die Anzahl der parallel eingenommenen (Dauer‑)Medikamente erhöhte das relative Risiko für die untersuchten Komplikationen unter Kontrolle für die anderen Faktoren im Modell nicht.

Bei vergleichender Analyse der relativen Risiken für die definierten Komplikationen zwischen Parkinson- und Vergleichskohorte, zeigten Parkinson-Patienten ein stark erhöhtes Risiko zu versterben (RR^ohne NK^ = 4,02 [95 %-CI 3,83–4,21]; RR^mit NK^ = 3,52 [95 %-CI 3,38–3,68]) oder an Halluzinationen (RR^ohne NK^ = 7,6 [95 %-CI 5,30–10,89]; RR^mit NK^ = 12,96 [95 %-CI 10,03–16,74]) zu leiden. Die Regressionsanalyse lieferte keine Hinweise für bestehende Stadt-Land-Unterschiede bezüglich der definierten Parkinson-spezifischen Komplikationen.

### Ambulante Versorgung

Die Untersuchung von Stadt-Land-Unterschieden ergab, dass Patienten in ländlich geprägten Regionen zu einem geringeren Anteil von Neurologen fachärztlich betreut wurden (Land: 78,4 %; Stadt; 84,4 %) und häufiger Parkinson-spezifische Medikamente vom Hausarzt verordnet bekamen (Land: 33,0 %; Stadt 20,4 %; *Zusatzmaterial 2, eTab. 7*). Spezielle Behandlungsformen, wie tiefe Hirnstimulation, Arzneimittelpumpen und multimodale Komplexbehandlung waren insgesamt selten, jedoch für Erkrankte mit Neurologenkontakt häufiger (Tab. 4; *Zusatzmaterial 2, eTab. 4*).

**Table 4:** 

Merkmal	Ohne NK (*n* = 21.666)	Mit NK (*n* = 45.782)	Insgesamt (*n* = 67.448)
Ambulante Versorgung			
Anteil Kontakt Hausarzt (%)	99,5	98,9	99,0
Anzahl Hausarztkontakte (MW (SD))	14,2 (8,1)	13,3 (7,6)	13,5 (7,7)
Anzahl alle Arztkontakte (MW (SD))	25,1 (19,6)	30,4 (16,7)	29,4 (17,4)

Angaben beziehen sich auf Merkmalsausprägungen pro Kalenderjahr im Beobachtungszeitraum 2011 bis 2019

*MW* Mittelwert, *NK* Neurologenkontakt im jeweiligen Jahr,* SD* Standardabweichung

**Figure 1:**
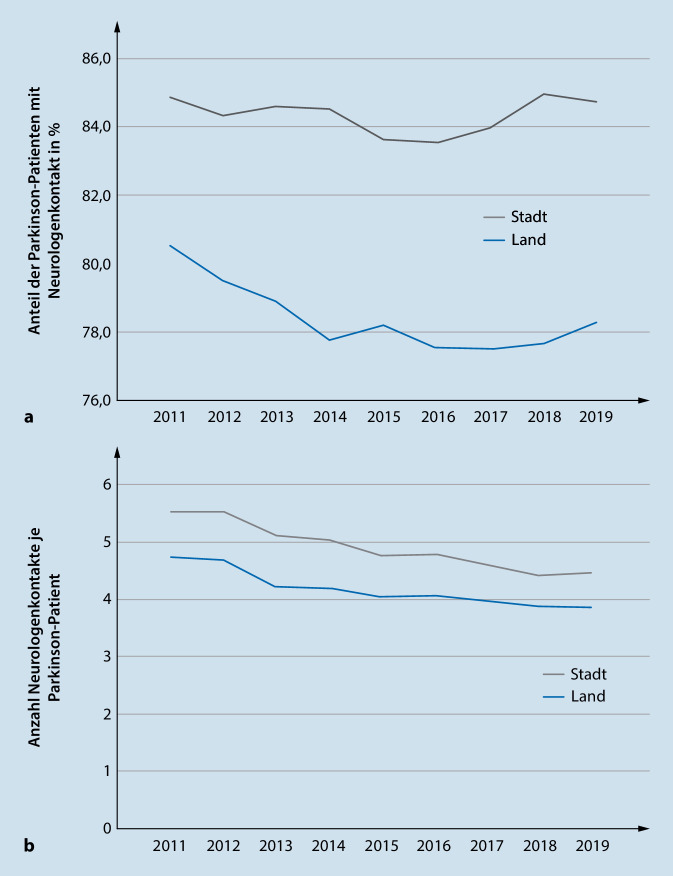

Die Untersuchungen möglicher Veränderungen über die Beobachtungszeit im Vergleich städtisch und ländlich geprägter Gebiete ergab, dass sowohl der Anteil der Parkinson-Patienten mit mindestens einem Hausarztkontakt (~99 %) als auch die durchschnittlichen Hausarztkontakte pro Jahr (~13) auf konstant hohem Niveau ohne erkennbaren zeitlichen Trend verliefen. Abb. 1 zeigt die Veränderung des Anteils der Parkinson-Patienten mit Neurologenkontakt (Abb. 1a) sowie die Anzahl Neurologenkontakte (Abb. 1b) im Beobachtungszeitraum 2011 bis 2019. In ländlich geprägten Gebieten ist insbesondere zwischen 2011 und 2015 ein abnehmender Anteil an Erkrankten mit Neurologenkontakt abzulesen. Zudem zeigt sich sowohl in städtisch als auch in ländlich geprägten Gebieten eine Abnahme der durchschnittlichen jährlichen Kontakte zum behandelnden Neurologen (Abb. 1b).

### Medikamentöse Parkinson-Therapie

In der vorliegenden dynamischen Kohorte wurden durchschnittlich mehr als 85 % der Parkinson-Patienten mit L‑Dopa behandelt. Neben dem anteiligen leichten Rückgang der Verordnungen von Dopaminagonisten und NMDA-Antagonisten ist ein zunehmender Anteil von Erkrankten abzulesen, die mit MAO-Inhibitoren versorgt wurden (Tab. 5). Eine Untersuchung regionaler Unterschiede ergab keine relevanten Unterschiede zwischen städtisch und ländlich geprägten Gebieten. Auffällig ist jedoch, dass bei Erkrankten mit behandelndem Neurologen der Anteil verschriebener Dopaminagonisten deutlich erhöht ist. Bei COMT- und MAO-Inhibitoren sind die Verordnungsanteile bei Erkrankten ohne behandelnden Neurologen über die Beobachtungszeit konstant, steigen jedoch bei Erkrankten mit Neurologen. Gleichzeitig ist der Anteil der Erkrankten unter Therapie mit Anticholinergika bei jenen mit Neurologenkontakt niedriger.

**Table 5:** 

Merkmal		2011–2013	2014–2016	2017–2019
L‑Dopa	Ohne NK	80,1	78,6	77,4
	Mit NK	86,4	87,0	87,9
Dopaminagonisten	Ohne NK	26,8	28,2	28,3
	Mit NK	52,8	49,2	46,4
COMT-Inhibitoren	Ohne NK	1,7	1,1	1,2
	Mit NK	2,4	2,1	5,2
MAO-Inhibitoren	Ohne NK	5,1	4,9	4,8
	Mit NK	13,1	15,6	17,8
NMDA-Antagonisten	Ohne NK	10,8	7,6	6,6
	Mit NK	17,1	13,9	11,2
Anticholinergika	Ohne NK	11,1	10,4	9,0
	Mit NK	5,1	4,7	4,1

Details siehe Zusatzmaterial 2, eTab. 5

*NK* Neurologenkontakt im jeweiligen Jahr

#### Heilmittelversorgung

Die Heilmittelversorgung über den Beobachtungszeitraum von 2011 bis 2019 ist in Tab. 6 aufgeführt. Demnach erhielten knapp zwei Drittel der eingeschlossenen Parkinson-Patienten – bei leicht ansteigendem Anteil über den Beobachtungszeitraum – eine Physiotherapie. Prozentual am stärksten zugenommen hat der Anteil der Parkinson-Patienten mit Verordnung von Ergotherapie (davon 80 % mit Indikation Zentralnervensystem) und Logopädie (fast ausschließlich für Störungen der Sprechmotorik und Schluckstörungen).

**Table 6:** 

Merkmal		2011–2013	2014–2016	2017–2019
Physio – unabhängig von Indikation	Ohne NK	43,7	47,4	48,9
	Mit NK	63,5	66,0	68,2
KG (Einzeln) – mI ZN2	Ohne NK	16,2	17,1	14,2
	Mit NK	31,0	32,0	27,8
Ergo – unabhängig von Indikation	Ohne NK	7,1	9,1	11,0
	Mit NK	10,5	13,8	17,0
Logo – unabhängig von Indikation	Ohne NK	2,9	4,1	4,3
	Mit NK	5,2	6,5	8,1

Details siehe Zusatzmaterial 2, eTab. 6

*mI* mit Indikation, *Ergo* Ergotherapie, *KG* Krankengymnastik, *Logo* Logopädie, *NK* Neurologenkontakt im jeweiligen Jahr,* Physio* Physiotherapie,* ZN2* Erkrankungen des Nervensystems einschl. des Rückenmarks nach Vollendung des 18. Lebensjahres

Insgesamt war der Anteil der Parkinson-Patienten mit Heilmittelversorgung aus städtisch geprägten Gebieten höher *(Zusatzmaterial 2, eTab. 6)*. Im Falle der speziellen Krankengymnastik und der Ergotherapie bestand ein über die Beobachtungszeit abnehmender Stadt-Land-Unterschied bei Parkinson-Patienten mit Neurologenkontakt. Der Anteil der Erkrankten mit Heilmittelversorgung war durchgängig bei jenen mit Neurologenkontakt höher.

## Diskussion

Mit dem bundesweit größten Anteil an Menschen über 70 Jahren (19,6 %)^3^ sind die Auswirkungen des demographischen Wandels bereits heute in Sachsen spürbar. Die vorliegende Sekundärdatenanalyse nutzt umfangreiche Routinedaten und eine gematchte Vergleichskohorte, um detaillierte Aussagen zur Charakterisierung der Parkinson-Kohorte, zur Änderung der Inanspruchnahme über die Zeit sowie zu assoziierten Komplikationen der Parkinson-Krankheit zu treffen. Diese sind üblicherweise nicht Teil klinischer Studien, liefern jedoch relevante Informationen zur Versorgungspraxis (Tab. 3). Altersstruktur, vorliegende Begleiterkrankungen sowie Anzahl parallel eingenommener Medikamente deckten sich mit Ergebnissen zurückliegender Sekundärdatenanalysen [14, 19, 20]. Die über den Beobachtungszeitraum ansteigenden Fallzahlen bestätigten den aus epidemiologischen Studien bekannten Trend der steigenden Prävalenz von Parkinson-Patienten [11, 16].

In der Gesamtschau hatten Erkrankte der dynamischen Parkinson-Kohorte mehr Sterbefälle, mehr Begleiterkrankungen, häufigere Behandlungen im Krankenhaus, nahmen häufiger mehr als vier Dauermedikamente ein und es traten häufiger Komplikationen auf als in der gematchten Vergleichskohorte.

Deutliche Unterschiede wurden für die neurologische Facharztbehandlung festgestellt: Parkinson-Patienten ohne Neurologenkontakt wiesen eine erhöhte Mortalität auf, was zum Teil durch ein erhöhtes Durchschnittsalter erklärt werden kann. Ein über die Beobachtungszeit deutlich höherer Anteil verordneter Dopaminagonisten oder auch der steigende Anteil von Behandlungen mit COMT- und MAO-Inhibitoren bei Erkrankten mit behandelndem Neurologen zeigen Änderungen in der Versorgungspraxis auf und können ein Beleg für bestehende Versorgungsunterschiede sein. Darüber hinaus bestehen in allen untersuchten Heilmittelkategorien erhöhte Inanspruchnahmen bei Erkrankten mit Neurologenkontakt. Auch wenn die Unterschiede der Parkinson-spezifischen Komplikationen (außer Tod) zwischen den zwei Gruppen insgesamt gering waren, legen die beschriebenen Unterschiede mit Blick auf Mortalität, medikamentöse Parkinson-Therapie und Heilmittelversorgung nahe, dass eine innovative Parkinson-Therapie von der (Mit‑)Behandlung durch Neurologen abhängt. Vor dem Hintergrund von Leitlinienupdates und einem veränderlichen Spektrum verfügbarer Medikamente unterstreicht dies die Bedeutung und den Effekt von Fortbildungen zur Vermittlung Parkinson-spezifischer (Medikations‑)Kompetenz.

Regionale Unterschiede bestehen im Hinblick auf den Anteil von Parkinson-Patienten, die nicht durch Neurologen betreut wurden und in Folge – scheinbar kompensatorisch – häufiger Parkinson-spezifische Medikamente vom Hausarzt verordnet bekamen. Zudem ist die Zahl der Arztkontakte von Parkinson-Patienten zum Neurologen in städtisch geprägten Gebieten im Vergleich zu ländlichen erhöht. Auch wenn insgesamt lediglich geringe Unterschiede in der Inanspruchnahme zwischen städtisch und ländlich geprägten Gebieten erkennbar waren, belegen vergleichbare Sekundärdatenanalysen, dass Parkinson-Patienten aus ländlichen Regionen tendenziell später diagnostiziert werden und weniger Parkinson-spezifische Therapien in Anspruch nehmen als Erkrankte aus urbanen Gebieten [15, 18].

Querschnitterhebungen zeigen, dass, aus Sicht der Erkrankten, aktivierende Therapien, wie Physio‑/Ergotherapie und Logopädie, bisher nicht zufriedenstellend in die Versorgung integriert werden [3, 7]. Befragungsergebnisse legen nahe, dass 70 % der Parkinson-Patienten einen Unterstützungsbedarf im Bereich der Sprache und des Sprechens haben [12]. Vor diesem Hintergrund ist der beobachtete Anstieg an Ergotherapie und Logopädie über die Beobachtungszeit erfreulich; der tatsächliche Bedarf könnte jedoch weitaus höher liegen. Beispiele aus den Niederlanden belegen, dass die Inanspruchnahme spezialisierter Heilmittelerbringung von Parkinson-Patienten durch gezielte Vernetzung der Leistungserbringer innerhalb von fünf Jahren signifikant gesteigert werden konnte [5]. Auch vor diesem Hintergrund gelten regionale und interdisziplinäre Versorgungsnetzwerke als Schlüssel, um eine individualisierte und an der jeweiligen Erkrankungsphase ausgerichtete Parkinson-Therapie frühzeitig zu initiieren, krankheitsspezifische Expertise zu bündeln und die Anwendung nichtmedikamentöser Therapien zu stärken [6, 7, 21, 22].

### Limitationen

Durch den unmittelbaren zeitlichen und abrechnungsrelevanten Bezug ist von einem entsprechenden Detailgrad und einem hohen Maß an Vollständigkeit der genutzten Routinedaten auszugehen. Außerdem vereint die vorliegende Sekundärdatenanalyse verschiedene Sektoren der Leistungserbringung. Die Verwendung einer gematchten Vergleichskohorte ohne Bewegungsstörung erlaubt Rückschlüsse auf die zusätzliche Krankheitslast durch die Parkinson-Krankheit.

Aufgrund des Einschlusses der Parkinson-Patienten nach Vorliegen der Aufgreifkriterien (Tab. 1) ab dem Folgejahr, kann es jedoch zu einer geringfügigen Unterschätzung der Patientenzahlen gekommen sein. Umgekehrt wurde der Anstieg der Parkinson-Patienten etwas überschätzt, da Patienten, die erst in den späteren Jahren hinzukamen, aber nach 2019 Medikamente absetzten (Rechtszensierung), nicht identifiziert werden konnten. Der Schweregrad (z. B. mittels Hoehn-und-Yahr-Skala) konnte nicht abgebildet werden, da dies im ambulanten Bereich selten bzw. nur ungenau mit der Diagnose verschlüsselt wird. Um die Sensitivität der angewandten Falldefinition zu erhöhen, wurde eine Parkinson-Diagnose erst dann als hinreichend gesichert angesehen, wenn zusätzlich ein Parkinson-spezifisches Medikament verordnet wurde. Hintergrund sind die aus der Literatur bekannten Herausforderungen bei der Indikationsstellung sowie der – insbesondere in frühen Erkrankungsstadien – unsicheren Abgrenzung gegenüber anderen neurologischen Erkrankungen [31].

## Fazit für die Praxis

- Die vorgestellten Analysen können dazu beitragen, die komplexen Herausforderungen des demographischen Wandels datenbasiert abzubilden.
- Erkrankte der dynamischen Parkinson-Kohorte wiesen mehr Sterbefälle, mehr Begleiterkrankungen, häufigere Behandlungen im Krankenhaus und häufigere Komplikationen auf als jene der gematchten Vergleichskohorte (ohne ICD-10-Diagnose aus G20–26).
- Analysen über die Beobachtungszeit konnten Veränderungen der medikamentösen Parkinson-Therapie (Zunahme COMT- und MAO-Inhibitoren) sowie der Heilmittelerbringung (Zunahme Ergotherapie und Logopädie) abbilden. Von diesen Entwicklungen profitieren primär Erkrankte mit Neurologenkontakt.
- Die identifizierten Versorgungsunterschiede können helfen, innovative Versorgungskonzepte zur Bündelung interdisziplinärer Parkinson-spezifischer Kompetenzen zu entwickeln.

## Supplementary Information






## Footnotes

1 Aus Gründen der Lesbarkeit werden die männliche und weibliche Form synonym verwendet.

2 https://www.panos.care.

3 https://www-genesis.destatis.de/genesis/online (Stichtag: 31.12.2015); [Zugriff: 28.06.2021].
