# Supplementary material for: Treatment practice of patients with Parkinson’s disease in Saxony: A secondary data-based analysis of utilization in the observation period 2011–2019
Source: Nervenarzt. 2022 Mar 14;93(12):1206–18. [Article in German] doi: 10.1007/s00115-022-01273-7 (PMC9718707; doi:10.1007/s00115-022-01273-7)
Supplement: Supplementary file 1 [file 115_2022_1273_MOESM1_ESM.docx]

## Definitionen

Einteilung der Regionen

Für die Unterscheidung von städtisch- und ländlich-geprägtem Wohnsitz wurden die Großstädte Dresden, Leipzig, Chemnitz sowie deren Umlandgemeinden als Städte definiert, auch weil sich nur hier Kliniken der Maximalversorgung befinden.

**Tabelle 1 Regionale Aufteilung I – Stadt vs. Land**

| Region | PLZ-3 |
| --- | --- |
| Stadt | 010,011,012,013,014,041,042,043,044,091,092 |
| Land | 015, 016, 017, 018, 019, 026, 027, 028, 029, 046,047, 048, 079, 080, 081, 082, 083, 084, 085, 086, 093, 094, 095, 096 |


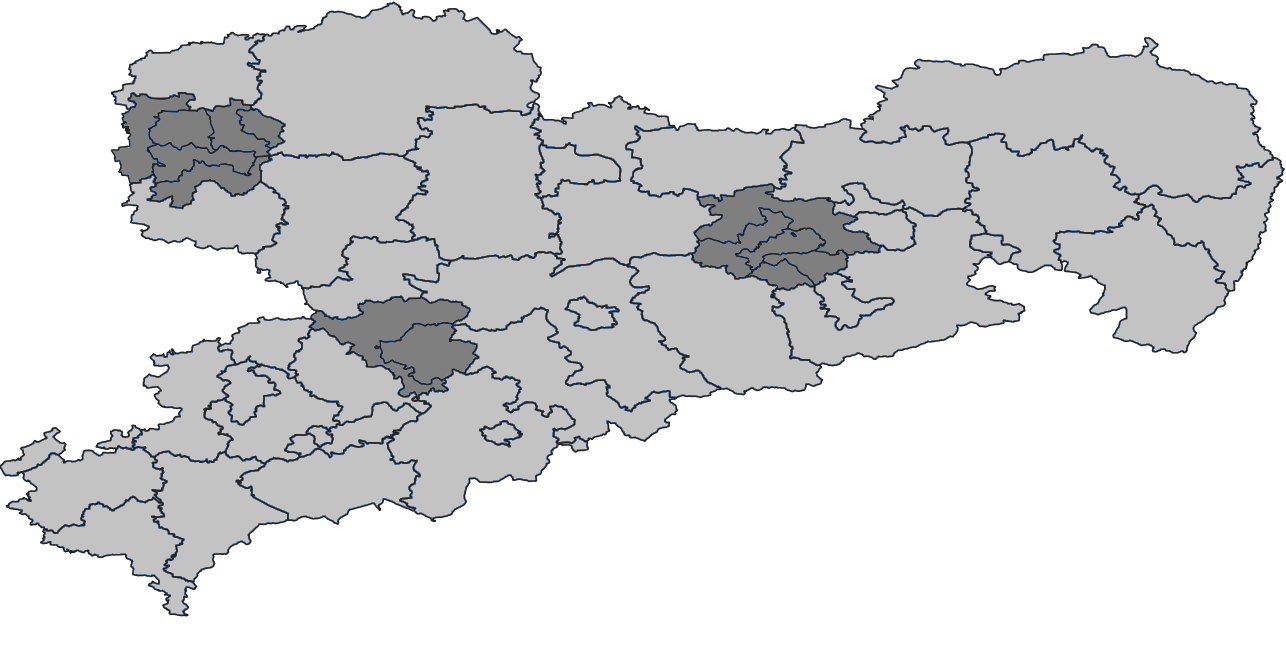


**Abbildung 1 Regionale Aufteilung – Stadt vs. Land (Karte)- Einteilung der Regionen in primär städtische (dunkelgrau) primär ländliche (hellgrau) Regionen anhand der 3-stelligen Postleitzahlen (PLZ)**

**Tabelle 2 Regionale Aufteilung II - Ost- vs. Westsachsen**

| Region | PLZ-3 |
| --- | --- |
| Ostsachsen | 010,011,012,013,014, 015, 016, 017, 018, 019, 026, 027, 028, 029 |
| Westsachsen | 042,043,044, 046, 047, 048, 079, 080, 081, 082, 083, 084, 085, 086, 091,092, 093, 094, 095, 096 |


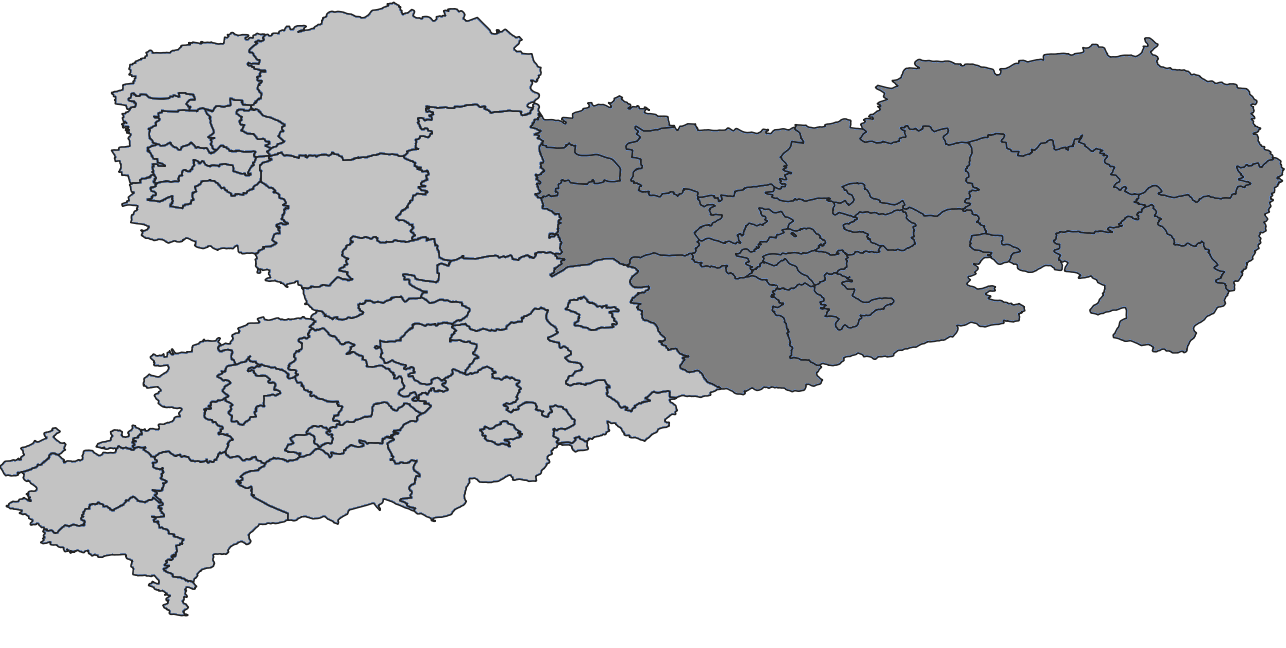


**Abbildung 1 Ostsachsen vs. Westsachsen - Einteilung in Ostsachsen (dunkelgrau) und Westsachsen (hellgrau) auf anhand der 3-stelligen PLZ-Bereiche**

Facharzt*innengruppen

Die Analysen der Facharzt*innenunterschiede erfolgen für folgende Facharzt*innengruppen der Hausärzte und Neurologen. Die Anzahl der Besuche unterschiedlicher Facharzt*innengruppen bezieht sich dabei ebenso auf die genannten Gruppen.

**Tabelle 3 Relevante Facharzt*innengruppen**

| ID | Fachgruppe | Arztnummer ab 2008/3 |
| --- | --- | --- |
| 1 | Hausarzt | 01,02,03 |
| 2 | Neurologe | 51, 53 |
| 3 | Sonstige | Alle anderen |

Inanspruchnahme der ambulanten Versorgung

**Tabelle 4 Ambulante Versorgung**

| **Var** | **Variable** | **Beschreibung** |
| --- | --- | --- |
| 1 | Hausarztkontakte innerhalb Jahresscheibe (N, %) | Datumsangaben mit EBM von Arztgruppe 1 |
| 2 | Neurologenkontakte innerhalb Jahresscheibe (N, %) | Datumsangaben mit EBM von Arztgruppe 2 |
| 3 | Alle Ärzte innerhalb Jahresscheibe (N, %) | Datumsangaben mit EBM von alle Arztgruppen |

Inanspruchnahme der stationären Versorgung

**Tabelle 5 Stationäre Versorgung**

| **Var** | **Variable** |
| --- | --- |
| 1 | Krankenhausaufenthalt |
| 2 | Verweildauer 1 in Tagen (Mittelwert inkl. 95%-KI, Median, SD) |
| 3 | Krankenhausaufenthalt wegen Parkinson als Hauptentlassdiagnose |
| 4 | Verweildauer 3 in Tagen (Mittelwert inkl. 95%-KI, Median, SD) |
| 5 | Notfalleinweisung wegen Parkinson als Hauptentlassdiagnose |
| 6 | Verweildauer 4 in Tagen (Mittelwert inkl. 95%-KI, Median, SD) |

Morbidität

**Tabelle 6 Krankheitsliste in Anlehnung an MultiCare-Studie**

| **Nr.** | **Kurzbeschreibung** | **ICD-10-Codes** |
| --- | --- | --- |
| 1 | Hypertonie/Bluthochdruck | I10–I15 |
| 2 | Störungen des Fettstoffwechsels | E78 |
| 3 | Chronische Rückenscherzen | M40–M45, M47, M48.0–M48.2, M48.5–M48.9 M50–M54 |
| 4 | Schwere Sehprobleme | H17–H18, H25–H28, H31, H33, H34.1–H34.2, H34.8–H34.9, H35–H36, H40, H43, H4, H54 |
| 5 | Arthrose | M15–M19 |
| 6 | Diabetes mellitus (alle Formen) | E10–E14 |
| 7 | Chronische ischämische Herzkrankheit | I20, I21, I25 |
| 8 | Krankheiten der Schilddrüse | E01–E05, E06.1–E06.3, E06.5, E06.9, E07 |
| 9 | Kardiale Arrhythmien | I44–I45, I46.0, I46.9, I47–I48, I49.1–I49.9 |
| 10 | Adipositas | E66 |
| 11 | Hyperurikämie/Gicht | E79, M10 |
| 12 | Prostatahyperplasie | N40 |
| 13 | Varizen der unteren Extremitäten | I83, I87.2 |
| 14 | Leberkrankheiten | K70, K71.3–K71.5, K71.7, K72.1, K72.7, K72.9, K73–K74, K76 |
| 15 | Depression | F32–F33 |
| 16 | Asthma bronchiale/COPD | J40–J45, J47 |
| 17 | Nichtentzündliche gynäkologische Probleme | N81, N84–N90, N93, N95 |
| 18 | Atherosklerose/PAVK | I65–I66, I67.2, I70, I73.9 |
| 19 | Osteoporose | M80–M82 |
| 20 | Niereninsuffizienz | N18–N19 |
| 21 | Z. n. Schlaganfall (inkl. TIA) | G45, I60–I64, I69 |
| 22 | Herzinsuffizienz | I50 |
| 23 | Schwerer Hörverlust | H90, H91.0, H91.1, H91.3, H91.8, H91.9 |
| 24 | Gallensteinleiden chronische Gallenblasenentzündung | K80, K81.1 |
| 25 | Somatoforme Störungen | F45 |
| 26 | Hämorrhoiden | K64 |
| 27 | Divertikulose des Darmes | K57 |
| 28 | Rheuma, chronische Polyarthritis | M05–M06, M79.0 |
| 29 | Herzklappenkrankheiten | I34–I37 |
| 30 | Neuropathien | G50–G64 |
| 31 | Schwindel | H81–H82, R42 |
| 32 | Demenz | F00–F03, F05.1, G30, G31, R54 |
| 33 | Harninkontinenz | N39.3–N39.4, R32 |
| 34 | Nieren-/Ureterstein | N20 |
| 35 | Anämie | D50–D53, D55–D58, D59.0–D59.2, D59.4–D59.9, D60.0, D60.8, D60.9, D61, D63–D64 |
| 36 | Angstzustände | F40–F41 |
| 37 | Psoriasis | L40 |
| 38 | Migräne/chronischer Kopfschmerz | G43, G44 |
| 39 | Bösartige Tumoren | C00–C14, C15–C26, C30–C39, C40–C41, C43–C44, C45–C49, C50, C51–C58, C60–C63, C64–C68, C69–C72, C73–C75, C81–C96, C76–C80, C97, D00–D09, D37–D48 |
| 40 | Allergien | H01.1, J30, K52.2, K90.0, L23, L27.2, L56.4, T78.1, T78.4, T88.7 |
| 41 | Chronische Gastritis/Refluxkrankheit | K21, K25.4–K25.9 K26.4–K26.9 K27.4–K27.9 K28.4–K28.9 K29.2–K29.9 |
| 42 | Sexuelle Dysfunktion | F52, N48.4 |
| 43 | Schlafstörungen | F51, G47 |
| 44 | Schwere Tabakabhängigkeit | F17 |
| 45 | Hypotonie | I95 |

*Krankheitsliste in Anlehnung an die MultiCare- Studie. ICD-10 für Hämorrhoiden wurde 2012 von I84 zu K64 geändert und hier entsprechend angepasst. Erkrankung 39 „Morbus Parkinson“ mit der ICD-10 Kodierung G20-22 wurde ersatzlos entfernt, da in der vorliegenden Analyse dies der primäre Untersuchungsgegenstand ist.*

Komplikationen

**Tabelle 7 Komplikationen**

| **Var** | **Variable** | **Beschreibung** |
| --- | --- | --- |
| 1 | Krankenhausaufenthalt (N, %) | Alle Aufenthalte wegen Parkinson  Notfalleinweisungen wegen Parkinson |
| 2 | Harnwegsinfekt (N, %) | Ambulant ICD-10: N39.0 |
| 3 | Frakturen (N, %)   - Alle Frakturen - Oberschenkelhalsfraktur | ICD10: S02, S12, S22, S32, S42, S52, S62, S72, S82, S92, T02  ICD10: S72.0 |
| 4 | Pneumonie N, %) | J16.x, J18.x, J69.0, J85.1 mit stationärer ODER ambulanter Behandlung mit Antibiotika (ATC J01), Antimykotika (ATC J02A) oder Virostatika (ATC J05) ODER Stationäre Hauptdiagnose A40.x-A41.x mit Nebendiagnose aus den anderen ICD-10. |
| 5 | Obstipation/Ileus (N, %) | ICD-10: K59.0, K56.0 (stationäre Hauptentlassdiagnose) |

Medikamentöse Parkinsontherapie

**Tabelle 8 Parkinsonspezifische Medikamente**

| **Var** | **Klasse lt. AVR** | **Anti-Parkinsonmedikament** | **ATC-Code*** |
| --- | --- | --- | --- |
| 1 | Levodopa (L-Dopa) | Levodopa | N04BA |
| 2 | Dopaminagonisten | Piribedil; Pramipexol; Lisurid;  Ropinirol; Apomorphin Cabergolin;  Rotigotin | N04BC |
| 3 | COMT-Inhibitoren | Entacapon; Tolcapon | N04BX |
| 4 | MAO-B-Hemmer | Selegilin;Rasagilin | N04BD |
| 5 | Anticholinergika | Biperiden; Bornaprin | N04AA |
| 6 | NMDA-Antagonist | Amantadin  Budipin | N04BB01  N04BX03 |
| 7 | Polypharmazie | - | Anzahl unterschiedlicher ATC-5 Steller in mindestens zwei von vier Quartalen |

*ATC- Kodierung nach Anatomisch-Therapeutisch-Chemisches Klassifikationssystem

Heilmittelversorgung

Für die Analyse von Heilmitteln als ambulante Therapie (Anzahl der Sitzungen im Kalenderjahr je Patienten mit Indikation) (N, %) wird neben dem spezifischen Heilmittel auch die überweisende Facharzt*innengruppe berücksichtigt (siehe Tabelle 3).

**Tabelle 9 Heilmittelversorgung**

| **Var** | **Verfahren** | **Heilmittelposition** |
| --- | --- | --- |
| 1 | Physikalische Therapie | X01- X20 |
| 2 | Physikalische Therapie mit Indikation WS | X01- X20 außer X020, X070, X080, X180, X190 |
| 3 | Krankengymnastik (Einzeln) mit Indikation ZN2 | X0301 – X0308, X0501- X0506 |
| 4 | Spezielle Krankengymnastik zur Behandlung von Erkrankungen des Zentralnervensystems mit Indikation ZN2 | X0710, X0711, X0712 |
| 5 | Kälte/Wärmetherapie mit Indikation ZN2 | X1501- X1534 |
| 6 | Ergotherapie mit Indikation EN2 | X4001- X4502 |
| 7 | Logopädie mit Indikation SC1 „Krankhafte Störungen Schluckakt“ | X3001- X3401 |
| 8 | Logopädie mit Indikation SP6 „Störungen Sprechmotorik“ | X3001- X3401 |

Hilfsmittelversorgung

**Tabelle 10 Hilfsmittelversorgung**

| **Var** | **Variable** | **Bereich** | **Beschreibung (Produktnummer)** |
| --- | --- | --- | --- |
| 1 | Selbstversorgung und Haushaltsführung | Notrufsysteme | 52.40.01 |
|  |  | Adaptationshilfen | 02.40.01./02./03./04./06.07. |
|  |  | Applikationshilfen | 03.36.04./10. |
| 2 | Pflegebetten | Pflegebetten | 50.45.01 |
|  |  | Pflegebettenzubehör | 50.45.02./03./04. |
|  |  | Pflegebetten, motorisch höhenverstellbar; Behindertengerechte Betten | 19.04.01 |
|  |  | Hilfsmittel gegen Dekubitus | 11. |
| 3 | Kommunikation | Hörhilfen | 13. |
|  |  | Sprechhilfen | 27.17. |
|  |  | Kommunikationshilfen | 16. |
|  |  | Sehhilfen | 25.21. |
| 4 | Pflegerische HM | Hilfsmittel bei Tracheostoma oder Laryngektomie | 12. |
|  |  | Inhalations- und Atemtherapiegeräte (ohne Verbrauchsmaterial) | 14.00./24. |
|  |  | Absauggeräte | 01.24.01./02 |
| 5 | Toiletten- und Inkontinenzhilfen | Saugende Bettschutzeinlagen | 51.40.014. |
|  |  | Einmal- und Ballonkatheter | 15.25.14./15. |
|  |  | Toilettensitzerhöhung, Toilettenstühle | 33.40. |
|  |  | Toilettenrollstühle (auch Dusch- und Toilettenrollstühle) | 18.46.01./02. |
|  |  |  |  |
|  |  | Urinflaschen | 51.40.011 |
|  |  | Bettbeutel mit Tropfkammer / Kombinierte Bett- und Beinbeutel mit Tropfkammer | 15.25.05/06/07 (Urin-Beinbeutel, Urin-Bettbeutel, Urinauffangbeutel für geschlossene Systeme) |
|  |  | saugende Bettschutzeinlagen | 51.40.014 |
|  |  | Einmal- und Ballonkatheter | 15.25.14/15 (Einmal- und Ballonkatheter) |
|  |  | Toilettensitzerhöhung, Toilettenstühle | 33.40. |
|  |  | Toilettenrollstühle (auch Dusch- und Toilettenrollstühle) | 18.46.01/02. |
|  |  | Urinflaschen | 51.40.011 |
| 6 | Mobilität | HM zur Kompressionstherapie | 17.06. |
|  |  | Orthesen/Schienen (und Korsagen) | 23. |
|  |  | Beinprothesen | 24. |
|  |  | Hand-/Gehstöcke, Gehgestelle, Unterarmstützen | 10.46.01 & 10.05.01/02/03 |
|  |  | Treppensteighilfen | 18.65.01 |
|  |  | Mobilitätshilfen - Lifter häuslicher Bereich | 22.40.01/02/05/06 |
|  |  | Rollatoren | 10.46.04. & 10.50.04./05 |
|  |  | Rollstuhl | 18.50.01./.02.03./04 & 18.51.0 & 18.99.01-12 |
|  |  | Mobilitätshilfen (Umsetz- und Hebehilfen) | 22.29.01.0./1./2./3./6 |
|  |  | Rampensysteme | 22.50. |
|  |  | Orthopädischer Maßschuh / Therapieschuh | 31.03.01./.02./03./04./05./06. |
|  |  | Einlagen | 08. |
| 7 | Bewegungs- und Schmerztherapie | Elektrostimulationsgeräte | 09. |
|  |  | therapeutische Bewegungsgeräte | 32. |
| 8 | Körperhygiene | Bade- und Duschhilfen | 04.40.01./02./03.05 & 18.46.03 |
|  |  | Waschsysteme (Kopf- oder Ganzkörper) | 51.45.01 |
|  |  | Halter/Greifhilfen für Produkte Körperhygiene | 02.40.25 |

Pflegbedürftigkeit

**Tabelle 11 Pflegebedarf entsprechend Pflegegrade und -stufen**

| **Var** | **Variable** | **Pflegestufe bis Ende 2016** | **Pflegegrad ab 01/2017** |
| --- | --- | --- | --- |
| 1 | Pflegebedarf entsprechend SGB XI (N, %) | Keine  1  2  3 | Kein  1  2  3  4  5 |
| 2 | Pflegeheim (N, %) |  |  |

Andere Behandlungen

**Tabelle 12 Andere Behandlungen nach OPS und EBM**

| Var | Variable | Abrechnung |
| --- | --- | --- |
| 1 | Tiefe Hirnstimulation | OPS 50282, 50283, 50285, 50286,50289, 5028a-c |
| 2 | Arzneimittelpumpe | OPS 8-97e |
| 3 | Multimodale Komplexbehandlung | OPS 8-97d |
| 4 | Verhaltenstherapie | EBM 35220-35225 bis 2017Q3, dann 35421-35425, 35543-35559 |

EBM- Einheitlicher Bewertungsmaßstab für ärztliche Leistungen im ambulanten Bereich

OPS - Operationen- und Prozedurenschlüssel für stationäre Verfahren
