# Supplementary material for: Treatment practice of patients with Parkinson’s disease in Saxony: A secondary data-based analysis of utilization in the observation period 2011–2019
Source: Nervenarzt. 2022 Mar 14;93(12):1206–18. [Article in German] doi: 10.1007/s00115-022-01273-7 (PMC9718707; doi:10.1007/s00115-022-01273-7)
Supplement: Supplementary file 2 [file 115_2022_1273_MOESM2_ESM.docx]

## Zuätzliche Tabellen

*eTabelle 1: Anteile der 4 Untersuchungsgruppen in den definierten Altersgruppen*

| Merkmal | Stadt | | Land | | Insgesamt |
| --- | --- | --- | --- | --- | --- |
| **Altersgruppen in %** | Ohne NK | Mit NK | Ohne NK | Mit NK |  |
| <60 | 2,8 | 3,7 | 3,1 | 4,3 | 3,9 |
| 60-69 | 6,7 | 10,0 | 6,9 | 11,5 | 10,2 |
| 70-74 | 8,6 | 13,3 | 8,3 | 13,8 | 12,6 |
| 75-79 | 17,8 | 22,4 | 17,4 | 24,7 | 22,6 |
| 80-84 | 25,1 | 24,3 | 25,9 | 25,1 | 25,0 |
| 85-89 | 23,8 | 17,4 | 24,1 | 14,8 | 17,3 |
| >89 | 15,3 | 8,8 | 14,3 | 5,8 | 8,4 |

**NK** Neurologenkontakt im jeweiligen Jahr

*eTabelle 2: Vergleich der Morbidität, Hilfsmittelversorgung und Komplikationen mit Vergleichskohorte*

| Merkmal | Parkinsonkohorte (n=67.448^¥^) | | | | | Vergleichskohorte (n=674.480^¥^) | |  |
| --- | --- | --- | --- | --- | --- | --- | --- | --- |
| **Morbidität** | **Stadt** | | **Land** | | | **Stadt** (n=216.660) | **Land** (n=457.820) |  |
|  | **Ohne NK**  (n=3.389) | **Mit NK**  (n=18.277) | **Ohne** **NK**  (n=9.891) | **Mit** **NK**  (n=35.891) | |  |  |  |
| Anzahl Dauermedikamente MW (SD) | 6,7 (3,8) | 6,9 (3,6) | 6,7 (3,7) | 6,8 (3,5) | | 4,4 (3,3) | 4,3 (3,3) | |
| Anteil Polypharmazie (≥5 Medikamente) in % | 71,2 | 73,9 | 72,1 | 72,6 | | 43,7 | 43,0 | |
| MultiCare (0-44) MW (SD) | 9,8 (4,5) | 10,6 (4,7) | 9,9 (4,6) | 10 (4,7) | | 9,0 (4,7) | 8,59 (4,7) | |
| Anteil mit Pflegestufe/-grad | 62,1 | 54,5 | 60,3 | 52,4 | | 21,5 | 21,0 | |
| **Hilfsmittelbereiche** | | | | |  | |  |  |
| Selbstversorgung u. Haushaltsführung in % | 5,6 | 4,3 | 4,6 | 3,5 | | 1,7 | 1,4 |  |
| Pflegebetten in % | 8,4 | 6,8 | 11,2 | 8,2 | | 1,9 | 2,6 |  |
| Kommunikation in % | 4,0 | 4,6 | 3,6 | 3,9 | | 4,9 | 4,3 |  |
| Pflegerische Hilfsmittel in % | 3,9 | 4,3 | 4,5 | 3,9 | | 2,8 | 2,8 |  |
| Toiletten- und Inkontinenzhilfen in % | 13,3 | 12,7 | 14,3 | 13,2 | | 5,2 | 5,8 |  |
| Mobilität in % | 30,5 | 37,7 | 31,3 | 36,2 | | 23,1 | 22,0 |  |
| Bewegungs- und Schmerztherapie in % | 1,0 | 1,8 | 1,1 | 1,8 | | 1,4 | 1,1 |  |
| Körperhygiene in % | 12,3 | 12,1 | 12,0 | 13,0 | | 4,8 | 5,4 |  |
| Insgesamt (unabh. der Kategorie) in % | 52,4 | 55,2 | 53,3 | 53,9 | | 34,3 | 33,5 |  |
| **Komplikationen** | | | | |  | |  |  |
| Tod in % | 18,5 | 11,9 | 17,7 | 10,4 | | 6,4 | 6,2 |  |
| Krankenhausaufnahmen in % | 43,8 | 46,2 | 45,0 | 46,5 | | 29,6 | 31,0 |  |
| Krankenhausaufnahmen in %* | 3,4 | 9,2 | 3,9 | 8,9 | | - | - |  |
| Krankenhausaufenthalt (Notfall) in % * | 1,6 | 2,7 | 1,5 | 2,5 | | - | - |  |
| Harnwegsinfekt in % | 10,3 | 9,6 | 8,7 | 8,6 | | 6,3 | 5,3 |  |
| Alle Frakturen in % | 4,6 | 4,5 | 4,2 | 4,0 | | 2,4 | 2,4 |  |
| Oberschenkelhalsfraktur in % | 0,9 | 0,8 | 1,1 | 0,8 | | 0,4 | 0,3 |  |
| Pneumonie ambulant in % | 3,5 | 2,5 | 2,7 | 2,0 | | 1,2 | 1,1 |  |
| Pneumonie stationär in % | 3,4 | 2,9 | 3,9 | 3,3 | | 1,1 | 1,3 |  |
| Aspirationspneumonie ambulant in % | 0,3 | 0,2 | 0,1 | 0,1 | | 0,02 | 0,03 |  |
| Aspirationspneumonie stationär in % | 0,6 | 0,6 | 0,7 | 0,5 | | 0,1 | 0,1 |  |
| Obstipation/Ileus in % | 1,7 | 1,4 | 1,5 | 1,1 | | 0,6 | 0,5 |  |
| Halluzinationen in % | 0,5 | 0,6 | 0,3 | 0,7 | | 0,0 | 0,0 |  |

Angaben beziehen sich auf Merkmalsausprägungen pro Kalenderjahr im Beobachtungszeitraum 2011-2019; Matching der Vergleichskohorte erfolgte entsprechend Alter, Geschlecht, Versicherungsjahr (2011-2019) sowie Stadt/Land; **^¥^** Angabe in Patientenjahren; ***** Merkmale beziehen sich auf Hospitalisierungen *wegen Mb. Parkinson*; **MW** Mittelwert; **N** Anzahl (absolute Zahlen); **NK** Neurologenkontakt im jeweiligen Jahr; **SD** Standardabweichung;

Häufigste Begleiterkrankungen entfallen in der Parkinsonkohorte auf die arterielle Hypertonie (82,1%), chronischen Rückenschmerz (56,6%) und Demenz (48%). In der Vergleichskohorte sind zwar arterielle Hypertonie (82,3%) und chronischer Rückenschmerz (50,7%) ebenso häufigste Begleiterkrankungen, von einer Demenz sind jedoch nur 20,2% betroffen. Der Anteil an Erkrankten mit Pflegegrad/-stufe ist bei jenen ohne Neurologenkontakt höher.

*eTabelle 3: Vergleich der Komplikationsraten von Parkinsonerkrankten mit vs. ohne Neurologenkontakt (jährlich gemittelt für 3 Jahre) im Beobachtungszeitraum 2011-2019*

| Merkmal | 2011 – 2013 | | 2014 - 2016 | | 2017 - 2019 | |
| --- | --- | --- | --- | --- | --- | --- |
| **Komplikationen** | **Ohne NK**  (n=3.975) | **Mit NK**  (n=17.193) | **Ohne NK**  (n=4.515) | **Mit NK**  (n=17.811) | **Ohne NK**  (n=4.790) | **Mit NK**  (n=19.164) |
| Tod in % | 19,5 | 11,5 | 17,8 | 11,3 | 16,6 | 10,0 |
| Krankenhausaufnahmen in % | 44,2 | 46,2 | 44,4 | 46,5 | 45,4 | 46,4 |
| Krankenhausaufnahmen in %* | 4,2 | 8,6 | 3,7 | 9,2 | 3,4 | 9,1 |
| Krankenhausaufenthalt (Notfall) in % * | 1,8 | 2,3 | 1,5 | 2,7 | 1,3 | 2,7 |
| Harnwegsinfekt in % | 9,3 | 8,6 | 9,4 | 9,2 | 8,7 | 9,0 |
| Alle Frakturen in % | 3,6 | 4,1 | 4,4 | 4,3 | 4,6 | 4,1 |
| Oberschenkelhalsfraktur in % | 1,0 | 0,8 | 0,9 | 0,7 | 1,1 | 0,8 |
| Pneumonie ambulant in % | 3,5 | 2,2 | 2,9 | 2,2 | 2,5 | 2,2 |
| Pneumonie stationär in % | 3,9 | 3,1 | 3,8 | 3,2 | 3,6 | 3,2 |
| Aspirationspneumonie ambulant in % | 0,2 | 0,2 | 0,2 | 0,1 | 0,1 | 0,1 |
| Aspirationspneumonie stationär in % | 0,7 | 0,5 | 0,7 | 0,7 | 0,7 | 0,5 |
| Obstipation/Ileus in % | 0,9 | 1,0 | 1,6 | 1,1 | 1,9 | 1,5 |
| Halluzinationen in % | 0,4 | 0,7 | 0,5 | 0,6 | 0,3 | 0,8 |

Angaben beziehen sich auf Merkmalsausprägungen pro Kalenderjahr im Beobachtungszeitraum 2011-2019 **^¥^** Angabe in Patientenjahren; ***** Merkmale beziehen sich auf Hospitalisierungen *wegen Mb. Parkinson*; **NK** Neurologenkontakt im jeweiligen Jahr

*eTabelle 4: Deskription ausgewählter Merkmale der ambulanten Versorgung von Erkrankten mit/ohne Neurologenkontakt im Stadt-Land-Vergleich*

| Merkmal | Stadt | | Land | | Insgesamt (n=67.448) |
| --- | --- | --- | --- | --- | --- |
| **Ambulante Versorgung** | Ohne NK  (n=3.389) | Mit NK  (n=18.277) | Ohne NK  (n=9.891) | Mit NK  (n=35.891) |  |
| Anteil Kontakt Hausarzt in % | 99,4 | 98,9 | 99,5 | 98,9 | 99,0 |
| Anzahl Hausarztkontakte MW (SD) | 14,5 (8,3) | 13,7 (8,0) | 14,1 (8,1) | 13,1 (7,4) | 13,5 (7,7) |
| Anzahl alle Arztkontakte MW (SD) | 26,8 (19,5) | 32,5 (16,6) | 24,6 (19,7) | 29,3 (16,6) | 29,4 (17,4) |
| **Medikamentenverordnung durch…** | | | | | |
| Neurologen in % | 0,6 | 98,0 | 1,0 | 97,0 | 78,4 |
| Hausärzte in % | 45,1 | 15,8 | 71,1 | 22,5 | 28,9 |
| Andere oder unklare Ärzte in % | 64,7 | 6,54 | 35,9 | 6,4 | 13,7 |
| **Spezielle Behandlungsformen** | | | | | |
| Tiefe Hirnstimulation N (in %) | 2 (0,06) | 32 (0,18) | 5 (0,05) | 73 (0,20) | 112 (0,17) |
| Arzneimittelpumpe N (in %) | 1 (0,03) | 28 (0,15) | 3 (0,03) | 45 (0,13) | 77 (0,11) |
| Multimodale Komplexbehandlung N (in %) | 34 (1,00) | 777 (4,25) | 122 (1,23) | 1.279 (3,56) | 2.212 (3,28) |
| Verhaltenstherapie | 3 (0,09) | 61 (0,33) | 17 (0,17) | 100 (0,28) | 181 (0,27) |

Angaben beziehen sich auf Merkmalsausprägungen pro Kalenderjahr im Beobachtungszeitraum 2011-2019; **MW** Mittelwert; **NK** Neurologenkontakt im jeweiligen Jahr; **SD** Standardabweichung

*eTabelle 5: Verordnete parkinsonspezifische Medikamente im Beobachtungszeitraum 2011-2019 (Anteil in % pro Parkinsonerkrankten mit mindestens einer Verordnung pro Jahr gemittelt für 3 Jahre)*

| Merkmal | | 2011 - 2013 | | | 2014 – 2016 | | | 2017 - 2019 | | |
| --- | --- | --- | --- | --- | --- | --- | --- | --- | --- | --- |
|  |  | Stadt | Land | Insg. | Stadt | Land | Insg. | Stadt | Land | Insg. |
| L-Dopa | Ohne | 83,1 | 79,0 | 85,3 | 81,5 | 77,6 | 85,3 | 79,8 | 76,6 | 85,8 |
|  | Mit | 86,5 | 86,4 |  | 86,9 | 87,1 |  | 88,4 | 87,6 |  |
| Dopamin-Agonisten | Ohne | 28,2 | 26,3 | 47,9 | 30,8 | 27,3 | 44,9 | 28,6 | 28,2 | 42,7 |
|  | Mit | 48,6 | 54,8 |  | 45,0 | 51,3 |  | 43,0 | 48,1 |  |
| COMT-Inhibitoren | Ohne | 1,0 | 2,0 | 2,3 | 0,6 | 1,3 | 1,9 | 1,1 | 1,3 | 4,4 |
|  | Mit | 2,5 | 2,4 |  | 2,1 | 2,1 |  | 4,8 | 5,4 |  |
| MAO-Inhibitoren | Ohne | 5,2 | 5,0 | 11,6 | 4,9 | 4,9 | 13,5 | 5,3 | 4,6 | 15,2 |
|  | Mit | 11,0 | 14,1 |  | 13,0 | 17,0 |  | 15,6 | 19,0 |  |
| NMDA-Antagonisten | Ohne | 10,1 | 11,0 | 15,9 | 6,7 | 7,9 | 12,6 | 7,0 | 6,5 | 10,2 |
|  | Mit | 17,3 | 17,0 |  | 13,6 | 14,1 |  | 10,1 | 11,7 |  |
| Anticholinergika | Ohne | 9,8 | 11,6 | 6,2 | 9,0 | 10,8 | 5,9 | 9,2 | 9,0 | 5,1 |
|  | Mit | 4,5 | 5,4 |  | 4,8 | 4,7 |  | 3,9 | 4,2 |  |

**Mit** Parkinsonerkrankte mit Neurologenkontakt im jeweiligen Jahr; **Ohne** Parkinsonerkrankte ohne Neurologenkontakt im jeweiligen Jahr

*eTabelle 6: Inanspruchnahme der Heilmittelversorgung (Anteil in % und Mittelwert der Behandlungseinheiten je Erkranktem mit Verordnung jährlich gemittelt für 3 Jahre) im Beobachtungszeitraum 2011-2019*

| Merkmal | | 2011 - 2013 | | | 2014 – 2016 | | | 2017 - 2019 | | |
| --- | --- | --- | --- | --- | --- | --- | --- | --- | --- | --- |
|  |  | Stadt | Land | Insg. | Stadt | Land | Insg. | Stadt | Land | Insg. |
| Physio - unabhängig von Indikation | Ohne | 45,7 | 43,0 | 59,8 | 50,1 | 46,4 | 62,2 | 50,6 | 48,4 | 64,4 |
|  | Mit | 66,3 | 62,1 |  | 68,1 | 64,9 |  | 68,4 | 68,1 |  |
| Physio – mI WS | Ohne | 13,2 | 13,2 | 19,6 | 14,8 | 13,8 | 19,4 | 15,7 | 15,2 | 18,6 |
|  | Mit | 22,0 | 20,6 |  | 22,8 | 19,7 |  | 22,0 | 18,1 |  |
| KG (Einzeln) - mI ZN2 | Ohne | 16,1 | 16,2 | 28,3 | 17,7 | 16,8 | 29,0 | 13,8 | 14,4 | 25,1 |
|  | Mit | 29,0 | 32,1 |  | 29,2 | 33,4 |  | 23,9 | 29,9 |  |
| Spezielle KG - mI ZN2 | Ohne | 5,6 | 3,6 | 6,4 | 4,6 | 4,0 | 6,8 | 5,9 | 4,6 | 8,6 |
|  | Mit | 9,0 | 5,9 |  | 9,1 | 6,7 |  | 9,6 | 9,4 |  |
| Ergo - unabhängig von Indikation | Ohne | 7,9 | 6,8 | 9,9 | 12,3 | 8,1 | 12,9 | 13,3 | 10,2 | 15,8 |
|  | Mit | 12,5 | 9,6 |  | 15,0 | 13,3 |  | 16,9 | 17,1 |  |
| Ergo - mI EN2 | Ohne | 4,5 | 4,6 | 7,6 | 6,3 | 5,5 | 10,1 | 6,9 | 6,2 | 12,2 |
|  | Mit | 9,6 | 7,6 |  | 11,9 | 10,9 |  | 13,1 | 13,9 |  |
| Logo - unabhängig von Indikation | Ohne | 3,7 | 2,6 | 4,8 | 5,5 | 3,6 | 6,0 | 5,0 | 4,0 | 7,3 |
|  | Mit | 6,5 | 4,6 |  | 8,1 | 5,6 |  | 9,5 | 7,3 |  |
| Logo - mI SC1 | Ohne | 0,9 | 0,9 | 1,2 | 2,3 | 1,0 | 1,7 | 2,1 | 1,5 | 2,6 |
|  | Mit | 1,5 | 1,2 |  | 2,3 | 1,6 |  | 3,2 | 2,7 |  |
| Logo mI SP5 | Ohne | 1,1 | 0,5 | 1,1 | 1,3 | 0,6 | 1,0 | 0,6 | 0,5 | 0,8 |
|  | Mit | 1,7 | 0,9 |  | 1,1 | 0,9 |  | 1,0 | 0,8 |  |
| Logo - mI SP6 | Ohne | 1,8 | 1,3 | 2,6 | 1,6 | 1,8 | 3,2 | 2,3 | 1,8 | 3,6 |
|  | Mit | 3,5 | 2,6 |  | 4,4 | 3,1 |  | 5,1 | 3,5 |  |

**EN2** Erkrankungen des Nervensystems; **Ergo** Ergotherapie; **KG** Krankengymnastik; **Logo** Logopädie; **mI** mit Indikation; **Mit** Parkinsonerkrankte mit Neurologenkontakt im jeweiligen Jahr; **MW** Mittelwert; **Ohne** Parkinsonerkrankte ohne Neurologenkontakt im jeweiligen Jahr; **Physio** Physiotherapie; **SC1** Krankhafte Störungen des Schluckaktes – Dysphagie; **SP5** Störungen der Sprache nach Abschluss der Sprachentwicklung; **SP6** Störungen der Sprechmotorik**; WS** Wirbelsäulenerkrankung; **ZN2** Erkrankungen des Nervensystems einschl. des Rückenmarks nach Vollendung des 18. Lebensjahrs

*eTabelle 7: Regressionsanalyse für das Auftreten verschiedener Komplikationen im Vergleich der dynamischen Parkinsonkohorte mit der Vergleichskohorte*

|  | Tod | Frakturen | Frakturen am Oberschenkelhals | Obstipation/Ileus | Pneumonie stationär | Pneumonie Ambulant | Harnwegsinfektion | Halluzinationen |
| --- | --- | --- | --- | --- | --- | --- | --- | --- |
| **Demographie** |  |  |  |  |  |  |  |  |
| Geschlecht (Referenz Frauen) | 1,52 (1,49-1,54) | 0,58 (0,56-0,60) | 0,78 (0,72-0,84) | 1,78 (1,68-1,89) | 2,27 (2,19-2,36) | 1,49 (1,43-1,56) | 0,61 (0,59-0,62) | 1,17 (0,99-1,39) |
| Altersgruppe <70 Jahre | 1 | 1 | 1 | 1 | 1 | 1 | 1 | 1 |
| Altersgruppe 70-79 Jahre | 1,96 (1,86-2,08) | 1,30 (1,21-1,39) | 2,20 (1,69-2,87) | 1,17 (1,02-1,34) | 1,41 (1,28-1,57) | 1,02 (0,94-1,12) | 1,16 (1,12-1,21) | 1,11 (0,80-1,55) |
| Altersgruppe 80-89 Jahre | 2,86 (2,71-3,03) | 1,66 (1,55-1,78) | 3,48 (2,67-4,52) | 1,09 (0,95-1,25) | 1,73 (1,56-1,91) | 1,23 (1,13-1,34) | 1,15 (1,11-1,20) | 1,02 (0,73-1,41) |
| Altersgruppe 90+ Jahre | 3,88 (3,66-4,12) | 2,03 (1,88-2,19) | 5,06 (3,85-6,66) | 0,90 (0,76-1,06) | 2,15 (1,93-2,41) | 1,68 (1,53-1,86) | 1,09 (1,04-1,14) | 0,85 (0,56-1,30) |
| **Morbidität** |  |  |  |  |  |  |  |  |
| Bis 5 Krankheiten | 1 | 1 | 1 | 1 | 1 | 1 | 1 | 1 |
| 6 bis 10 Krankheiten | 1,32 (1,28-1,35) | 1,54 (1,47-1,62) | 1,61 (1,41-1,85) | 3,44 (2,95-4,02) | 2,56 (2,35-2,79) | 1,80 (1,67-1,94) | 2,05 (1,98-2,12) | 1,57 (1,13-2,19) |
| 11 bis 15 Krankheiten | 1,96 (1,90-2,01) | 2,12 (2,02-2,23) | 2,25 (1,96-2,60) | 6,78 (5,80-7,93) | 4,29 (3,93-4,68) | 2,41 (2,22-2,61) | 3,15 (3,03-3,26) | 1,64 (1,15-2,35) |
| Mehr als 15 Krankheiten | 2,65 (2,56-2,74) | 2,57 (2,42-2,73) | 2,54 (2,16-2,99) | 10,51 (8,92-12,39) | 5,92 (5,39-6,51) | 3,36 (3,07-3,68) | 4,50 (4,32-4,68) | 2,68 (1,83-3,93) |
| Pflegestufe/Pflegegrad | 9,13 (8,91-9,35) | 3,65 (3,52-3,77) | 5,56 (5,03-6,14) | 6,43 (5,96-6,94) | 5,93 (5,63-6,24) | 2,76 (2,63-2,90) | 1,37 (1,34-1,40) | 7,09 (5,45-9,23) |
| **Medikation** |  |  |  |  |  |  |  |  |
| Bis 4 Dauermedikamente | 1 | 1 | 1 | 1 | 1 | 1 | 1 | 1 |
| 5 bis 7 Dauermedikamente | 0,30 (0,29-0,30) | 0,97 (0,93-1,00) | 0,89 (0,81-0,98) | 0,73 (0,68-0,79) | 0,67 (0,63-0,70) | 0,96 (0,91-1,01) | 1,04 (1,02-1,07) | 1,48 (1,14-1,92) |
| 8 bis 10 Dauermedikamente | 0,22 (0,22-0,23) | 0,92 (0,88-0,96) | 0,82 (0,73-0,91) | 0,74 (0,68-0,81) | 0,69 (0,65-0,73) | 1,01 (0,95-1,08) | 1,11 (1,08-1,14) | 1,30 (0,97-1,73) |
| Mehr als 10 Dauermedikamente | 0,18 (0,18-0,19) | 0,93 (0,88-0,98) | 0,68 (0,58-0,78) | 0,85 (0,77-0,94) | 0,79 (0,74-0,84) | 1,23 (1,14-1,32) | 1,22 (1,18-1,27) | 0,81 (0,57-1,17) |
| Rezepte für Parkinsonmedikation je 10 | 0,31 (0,28-0,34) | 0,91 (0,85-0,98) | 0,93 (0,77-1,11) | 0,67 (0,55-0,80) | 0,90 (0,80-1,00) | 1,06 (0,93-1,20) | 1,11 (1,05-1,18) | 1,55 (1,32-1,83) |
| DDD für Parkinsonmedikation je 100 | 0,93 (0,91-0,94) | 1,02 (1,01-1,03) | 1,00 (0,97-1,03) | 1,00 (0,97-1,03) | 0,94 (0,92-0,96) | 0,94 (0,91-0,96) | 0,98 (0,97-0,99) | 0,98 (0,95-1,00) |
| **Zielvariablen** |  |  |  |  |  |  |  |  |
| Kontrolle ohne Parkinson | 1 | 1 | 1 | 1 | 1 | 1 | 1 | 1 |
| Parkinson ohne ambulanten Neurologen | 4,02 (3,83-4,21) | 1,06 (0,97-1,16) | 1,76 (1,44-2,14) | 1,85 (1,55-2,20) | 2,02 (1,81-2,25) | 1,83 (1,61-2,07) | 1,22 (1,15-1,30) | 7,60 (5,30-10,89) |
| Parkinson mit ambulanten Neurologen | 3,52 (3,38-3,68) | 1,14 (1,06-1,22) | 1,61 (1,37-1,89) | 1,55 (1,35-1,77) | 2,03 (1,86-2,21) | 1,57 (1,42-1,73) | 1,20 (1,15-1,26) | 12,96 (10,03-16,74) |
| Wohnsitz Stadt (Referenz Land) | 0,98 (0,97-1,00) | 0,99 (0,96-1,02) | 1,06 (0,98-1,15) | 1,06 (1,00-1,13) | 0,82 (0,79-0,86) | 1,02 (0,98-1,06) | 1,12 (1,10-1,14) | 1,20 (1,00-1,43) |

Dargestellt sind einzelne Poisson Modelle mit robusten Standardfehlern unter Verwendung der 15.744 Patienten mit Parkinson in 67.448 Patientenjahren und 10mal so viel Kontrollpatienten ohne G2x Diagnosen im Zeitraum und exakt auf Geburtsjahr, Geschlecht, Wohnregion gematcht für das Auftreten verschiedener Komplikationen (Kopfzeile) in Abhängigkeit multipler Merkmale (Spalte links) in Relativen Risiken mit 95% Konfidenzintervall.
